# Cardiac Changes after Lactate-Guided Conditioning in Young Purebred Arabian Horses

**DOI:** 10.3390/ani13111800

**Published:** 2023-05-29

**Authors:** Maíra M. Santos, Gabriel V. Ramos, Isabela M. de Figueiredo, Tainá C. B. V. Silva, José C. Lacerda-Neto

**Affiliations:** Department of Clinic and Veterinary Surgery, São Paulo State University (Unesp), School of Agricultural and Veterinarian Sciences, Jaboticabal 14884-900, Brazil; maira_moreira04@yahoo.com.br (M.M.S.);

**Keywords:** athlete’s heart, cardiology, cardiovascular adaptation, echocardiography, equines, exercise physiology

## Abstract

**Simple Summary:**

Horses can develop “athlete’s heart” in a similar way to humans and dogs after physical training. The purpose of this study was to assess by echocardiography the cardiac changes of young purebred Arabian horses after performing a lactate-guided conditioning program. The conditioning program was established individually, according to the physical fitness of each animal, as determined by an incremental exercise test (IET). The IET and echocardiograms were carried out before and after six weeks of treadmill conditioning. At the end of the conditioning period, the horses showed improved aerobic capacity and structural and functional heart changes associated with “athlete’s heart”.

**Abstract:**

Cardiac adaptation to conditioning in horses was evaluated after empirical training based on trainers’ experience. Twelve purebred Arabian horses, aged (mean ± SD) 28.42 ± 3.75 months, which did not perform any type of exercise prior to the research, were submitted to treadmill conditioning for six weeks. The conditioning program was based on the velocity run by the horse at which the blood lactate concentration, determined in an incremental exercise test (IET), reached 2 mmol/L (V_2_). The velocity at which the blood lactate concentration reached 4 mmol/L (V_4_) was also determined. The echocardiograms were performed at rest with pulsed-wave and tissue Doppler imaging in B- and M-modes. All procedures were carried out before and after the conditioning period. The results showed increases in V_2_ (from 5.2 ± 0.3 to 6.7 ± 0.4 m/s) and V_4_ (from 5.8 ± 0.4 to 7.6 ± 0.5 m/s) (*p* < 0.0001). There were also increases in the left ventricle internal diameter at diastole (LVIDd), left ventricle mass (LV mass), and stroke volume (SV), while no changes were observed in the LV free wall thickness and mean and relative wall thicknesses. The conditioning protocol, which was completed by all horses, proved to be safe and efficient, as it improved the aerobic capacity of the animals. Finally, the cardiac remodeling that occurred was mainly associated with the effect of physical training.

## 1. Introduction

Cardiac remodeling in horses can be assessed by echocardiography, a procedure that accurately identifies training-associated changes [1,2,3,4,5]. Long-term intensive training leads to a cardiac adaptation known as “athlete’s heart”, which comprises two distinct morphologies [6]. In horses, the most common change found is eccentric hypertrophy, characterized by an increased left ventricular volume and the proportional increase in the myocardial wall thickness, thus maintaining the relative thickness. This adaptation observed in racing and endurance horses resembles the heart of human athletes who perform aerobic sports [2,3,4,7]. Concentric hypertrophy, on the other hand, is determined by wall thickening, relatively small ventricular volume, and increased relative thickness [6]. However, an increase in both ventricular diameter and relative wall thickness has been detected in thoroughbred horses, suggesting that these animals can also develop mixed hypertrophy [8].

Improvements in aerobic capacity induced by conditioning are largely related to changes in heart morphology and stroke volume (SV), which mainly occur in the first few months of training [9]. Nevertheless, similar to what is observed in human athletes, some studies on the effects of conditioning in horses showed varied results in structural and functional cardiac indexes. This was explained by the different characteristics of the training program, as well as its intensity, volume, and duration [2,6,7,8,10,11,12,13]. Additionally, disparities in body size, age, gender, and possibly between breeds make it difficult to extrapolate results and comparisons [2,5,8,14,15,16]. 

In human athletes, the “athlete’s heart” is extensively investigated in order to distinguish it from pathological conditions [17]. However, despite numerous studies showing the relationship among athletic performance, left ventricular diameter, and left ventricle mass in horses, there is still no detailed understanding of the cardiac structure and function in affected horses. Moreover, considering that these animals may have more homogeneous changes in their physical conditioning than humans because of the genetic selection for a particular sport, it is still necessary to investigate whether the cardiac adaptation induced by conditioning can assist in the selection of animals with greater athletic potential [16]. 

Although some studies employed echocardiography to evaluate the effect of conditioning in horses submitted to track [1,2,4,7,8,9,11] or treadmill training [10], they did not report the use of any objective method to guide the conditioning. So far, only one study, more specifically a doctoral thesis, investigated cardiac adaptation after lactate-guided treadmill conditioning. However, as the horses used were kept inactive prior to the research, potentially being considered detrained, this may have contributed to the absence of significant changes, except for a tendency towards hypertrophy in the studied period [12]. 

To the best of our knowledge, no research on heart changes resulting from conditioning guided by physiological parameters has been carried out using untrained horses. Blood lactate concentration is an indicator of anaerobic energy metabolism and has been used to assess the fitness of horses and guide conditioning intensity [18,19]. The velocity–blood lactate relationship helps to determine the conditioning load [20]. Thus, the prescription of conditioning load based on physiological parameters such as blood lactate concentration is essential to improving the performance of horses and avoiding sports injuries [20,21]. 

Therefore, this study aimed to assess by echocardiography the cardiac changes in young untrained horses. The animals, which did not perform any type of controlled exercise prior to the research, were submitted to a lactate-guided conditioning program for six weeks. We hypothesized that the prescribed conditioning method would increase the aerobic capacity of the horses and lead to a cardiac adaptation known as “athlete’s heart”.

## 2. Materials and Methods

### 2.1. Animals

The experimental procedures were approved by the Ethics Committee on the Use of Animals of the School of Agricultural and Veterinary Studies (FCAV) of São Paulo State University (UNESP), protocol no. 012601/19. A total of 12 naive purebred Arabian horses (nine females and three geldings), aged (mean ± SD) 28.42 ± 3.75 months, were used. The animals belonged to breeders who allowed the application of the proposed conditioning program. They were housed in the equine breeding area of the School of Agricultural and Veterinary Studies of UNESP, vaccinated against influenza, eastern/western encephalomyelitis, and tetanus (Fluvac Innovator^®^ EWT, Zoetis, Sao Paolo, Brazil), and dewormed every three months (Equest^®^ Pramox, Zoetis, Sao Paolo, Brazil). The horses were kept grouped together on a 100 × 70 m^2^ Tifton 85 grass paddock provided with water ad libitum. During the conditioning period, they were fed coast cross hay corresponding to 2% of body weight (BW) and supplemented with concentrate (1% of BW) divided into two daily portions. The nutrients and energy needed were provided according to the horses’ growth and moderate exercise requirements [22]. The supplementation was introduced fifteen days before the training period.

**Figure 1:**
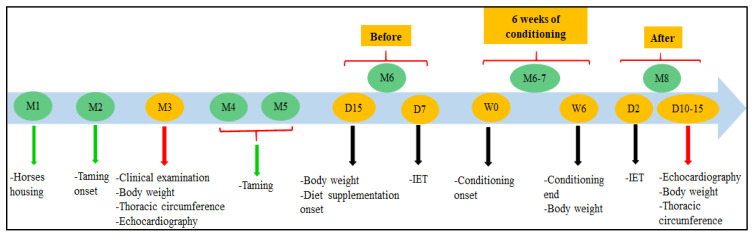
Experiment timeline. The naïve horses were housed at the university and submitted to rational taming. During this period, the experimental procedures were carried out. The incremental exercise test (IET) was performed before and after six weeks of conditioning. The echocardiographic images were obtained before and after the 5.5 month interval, including the conditioning period. M, month; D, day; W, week.

The physical and cardiovascular system examinations were performed to confirm if the horses were healthy. Cardiac auscultation did not detect significant murmurs or arrhythmias. BW was measured using a scale, while height and thoracic circumference were determined using a tape measure. BW was measured four times: in the first and second echocardiographic examinations, which had an interval of 5.5 months, and at the beginning and end of the conditioning period, which lasted six weeks. Between the first echocardiographic examination and the beginning of the conditioning period, the animals were submitted to rational taming, which was performed without physical exercise to facilitate handling in a horse stock and on a treadmill (Figure 1). Adaptation to treadmill exercise was carried out over three consecutive days and consisted of different speeds so that the horses could develop all gaits. This included 1 min at an inclination of 0% and 1 min at an inclination of 3% for each speed, i.e., 1.6, 3.4, 6, and 1.6 m/s.

### 2.2. Incremental Exercise Test (IET)

The incremental exercise test and physical training were performed on a treadmill (Galloper^®^, Sahinco, Palmital, Sao Paolo, Brazil) housed at the Equine Sports Medicine Laboratory of UNESP, Jaboticabal, Brazil, where the animals were previously adapted to the equipment and laboratory environment. First, the IET was used to determine the conditioning level of the horses and establish the individual training program. After six weeks of training, the same IET protocol was applied to assess the conditioning evolution. During the tests and training programs, the temperature of the treadmill room was maintained at 20 °C.

**Figure 2:**
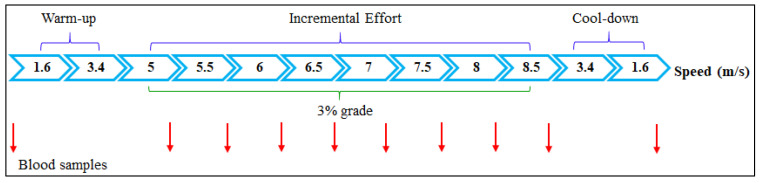
Schematic representation of the incremental exercise test (IET). The IET was performed before and after the conditioning period to determine the velocity run by the horse at which the blood lactate concentration reached 4 mmol/L (V_4_). The horses remained at each speed for 5 min. At the end of each speed of the incremental effort stage, the treadmill was turned off for 1 min for blood sample collection. After obtaining the lactate–velocity curve, it was possible to determine the velocity at which the blood lactate concentration reached 2 mmol/L (V_2_).

The IET consisted of three stages: warm-up, incremental effort, and cool-down. The horses remained at each speed for 5 min. During the warm-up, the speeds were 1.6 and 3.4 m/s at an inclination of 0%. Then, the inclination angle of the treadmill was increased to 3% and the speed was adjusted to 5 m/s, followed by speed increments of 0.5 m/s until a maximum speed of 8.5 m/s. At the end of this stage, the cool-down was performed at a speed of 3.4 and 1.6 m/s and an inclination of 0%. Between each speed of the incremental effort stage, the treadmill was turned off for 1 min (including the time to stop) for blood sample collection (Figure 2). The maximum velocity of each horse was the speed at which the blood lactate concentration reached or exceeded 4 mmol/L (V_4_), ending the incremental effort stage [21]. The horses were submitted to this IET before the training program and two days after its end. 

### 2.3. Blood Sample Collection and Laboratory Analysis

Prior to the IET, the horses were catheterized with a 14G central venous catheter (Biomedical^®^, Equipamentos e Produtos Médico-Cirúrgicos Ltda., Sao Paolo, Brasil) in the left jugular vein. An extender tube was attached to this catheter, and in order to maintain the access, the system was filled with heparinized solution. Blood samples of 4 mL were collected and placed in vacuum tubes containing EDTA and sodium fluoride (Becton Dickinson Indústrias Cirúrgicas LTDA, MG, Brasil) until analysis. Before collection, 15 mL of blood was removed and discarded to clean the system. 

Blood samples were collected at rest, before catheterization, directly from the jugular vein, while other samples were obtained from the extensor tube at the end of each speed of the incremental effort stage (when the treadmill stopped) and at the end of the cool-down. Immediately after collection, the blood lactate concentration was measured using an electroenzymatic methodology (Yellow Springs Instrument^®^, YSI 2300, Yellow Springs, OH, USA). After the determination of the venous blood lactate concentration, the samples were refrigerated until centrifugation for plasma separation (1500× *g* for 10 min), which occurred within 30 min of collection. At the end of the IET, the lactate concentration was measured in the refrigerated fresh plasma. The blood lactate concentration was used to determine the maximum velocity at which each animal should run during the IET (i.e., the velocity at which the lactate concentration was ≥4 mmol/L). In addition, the lactate concentration obtained in the plasma was used to establish the physical conditioning level of the horse and the conditioning program.

### 2.4. Conditioning Program

The intensity of the conditioning program was established based on V_2_, i.e., the velocity at which the plasma lactate concentration reached 2 mmol/L throughout the IET [21]. From the plasma lactate values determined in the incremental effort stage, it was possible to obtain the lactate–velocity curve, in which the exponential regression analysis was applied to determine the V_2_ of each horse. The physical training was carried out for six weeks and consisted of 5 sessions, each with 14 days, totaling 15 sessions. During the week, the animals rested for one day between the sessions.

**Figure 3:**
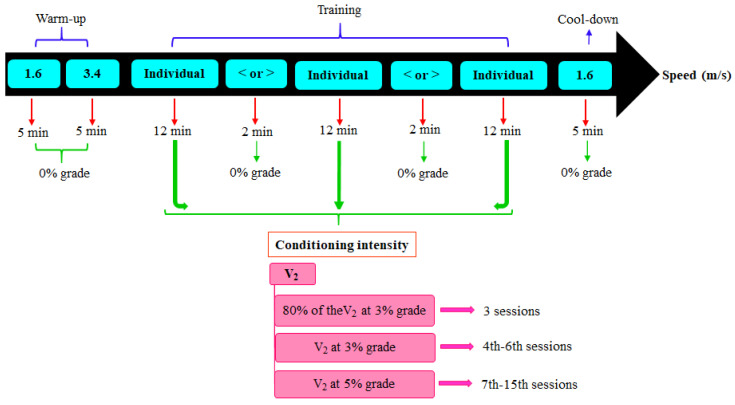
Schematic representation of the conditioning program performed on the treadmill. The intensity of the conditioning program was established based on V_2_ (velocity at which the plasma lactate concentration reached 2 mmol/L during the incremental exercise test). The conditioning intensity was increased every three sessions. Each session consisted of the following stages: warm-up, training, and cool-down. The training speeds were individualized, and to avoid repetitive strain injury, they were interspersed with 2 min of speed reduction or increase up to the sixth session. From the seventh session onwards, the horses cantered at the training speed, interspersed with 2 min of speed reduction to trot.

In the first three sessions, the treadmill training intensity was 80% of the individually calculated V_2_ at an inclination of 3%. From the fourth to the sixth session, the animals were trained at 100% of the V_2_ and an inclination of 3%. From the seventh session onwards, the inclination was increased to 5%. In each session, the horses exercised for 55 min, with 10 min of warm-up (5 min at 1.6 m/s and 5 min at 3.4 m/s at 0% inclination), 40 min of training, and 5 min of cool-down (1.6 m/s at 0% inclination). To avoid repetitive strain injury, the 40 min training was divided and interspersed until the sixth session, as follows: 12 min at the speed and inclination described above and 2 min of canter at an inclination of 0% and the lowest possible speed, which was adjusted on an individual basis. From the seventh session onwards, the horses cantered at the training speed, interspersed with 2 min of trotting at 4 m/s and 0% inclination (Figure 3). Two days after the end of the six weeks of training, the animals were submitted to the IET to determine the level of conditioning through V_4_ (determined from plasma lactate concentration).

### 2.5. Echocardiography

The echocardiographic examination was performed at the Equine Sports Medicine Laboratory using a portable device (Esaote^®^, MyLab Alpha, Genova, Italy), a 1–4 MHz probe, and simultaneous electrocardiogram. Firstly, the animals were acclimatized for 15 min in a climate-controlled room (20 °C) before examination. Then, they were kept at rest and in a horse stock without sedation. Afterwards, the animals had their right and left parasternal regions shaved for the application of the transmission gel. The standardized images were obtained in B- and M-modes using pulsed-wave and tissue Doppler imaging. Initially, the qualitative assessment of the cardiac structures and their dimensions was performed in the right parasternal window in B-mode and real time [23]. The measurements were made offline based on the images and videos recorded. The average of three cardiac cycles was obtained, excluding cycles containing blocks and the following cycle. The same examiner (MMS) performed all echocardiographic examinations and measurements. All measurements were made according to the inner edge and leading edge-to-leading edge method in B- and M-modes, respectively [23]. The examination of the untrained animals was carried out before the IET. After the conditioning program, images were acquired between 10 and 15 days, during which the animals remained in the stall.

### 2.6. B-Mode Measurements

The maximum diameter of the left atrium (LA) was obtained at the end of systole, one frame before the mitral valve opening, from the 4-chamber long-axis image focusing on the LA in the right parasternal window, while the diameter of the aorta artery (Ao) was measured at the end of diastole in the sinus of Valsalva from the image of the left ventricle (LV) outflow tract before the QRS. From these measurements, it was possible to calculate the LA/Ao ratio [24]. To determine the stroke volume, the aortic diameter was measured in the valve annulus at the peak of systole [25]. 

### 2.7. M-Mode Measurements

The LV measurements were performed in the right parasternal window from the short-axis of the LV to the chordal level. End-diastolic measurements were obtained immediately before the QRS complex, whilst systolic measurements were taken at the contraction peak, i.e., in the smallest ventricular lumen. The following values were measured: end-diastolic interventricular septum thickness (IVSd), end-systolic interventricular septum thickness (IVSs), end-diastolic left ventricular internal diameter (LVIDd), end-systolic left ventricular internal diameter (LVIDs), end-diastolic left ventricular free wall thickness (LVFWd), and end-systolic left ventricular free wall thickness (LVFWs) [26]. The values of LV mean wall thickness (MWT), relative wall thickness (RWT), LV mass [2], fractional shortening (FS) [26], and ejection fraction (EF) [27] were calculated (Table S1). To calculate the EF, the volumes were determined by the equation modified by Teichholz et al. [28]. 

### 2.8. Pulsed Doppler Measurements

Measurements of the transmitral and aortic flows were performed in the left parasternal window [26]. From the transmitral flow it was possible to measure the peak E (E_max_) and A (A_max_) wave velocities and calculate the E_max_-to-A_max_ ratio (E_max_:A_max_) [29], while from the aortic flow it was possible to obtain the pre-ejection period (PEP), the ejection time (ET), the PEP-to-ET ratio (PEP/ET), the time to peak (TTP), the maximal velocity of the aortic flow (V_max_), and the velocity time integral (VTI). Afterwards, the following values were determined: ejection time index (ETI) corrected to heart rate (HR), stroke volume (SV), cardiac output (CO), stroke index (SI), and mean velocity of circumferential fiber shortening (Vcf) (Table S1) [25]. 

The tissue Doppler imaging was performed with the region of interest in the LVFW in the short-axis of the LV, in the right parasternal window. The following values were then obtained: PEP, ET, PEP/ET, isovolumic relaxation time (IVRT), early (Em) and final (Am) diastolic velocities, and ejection velocity (Sm) [24]. 

### 2.9. Statistical Analysis

All analyses were carried out using Rstudio software (v. 4.2.1) at a significance level of 5%. The data were evaluated using the Shapiro–Wilk normality test and expressed as the mean and standard deviation. The paired t-test was applied to determine differences before and after the training program. To establish the relationship between the measurements and some cardiac indices, body weight, and thoracic circumference, the Pearson’s correlation coefficient was verified. Principal component analysis was used in order to reduce data dimensionality and understand the behavior of each variable in relation to the principal components. To decide the number of factors, the Kaiser criterion was adopted, which suggests that the choice of factors takes into account eigenvalues equal to or greater than 1 [30]. 

## 3. Results

**Figure 4:**
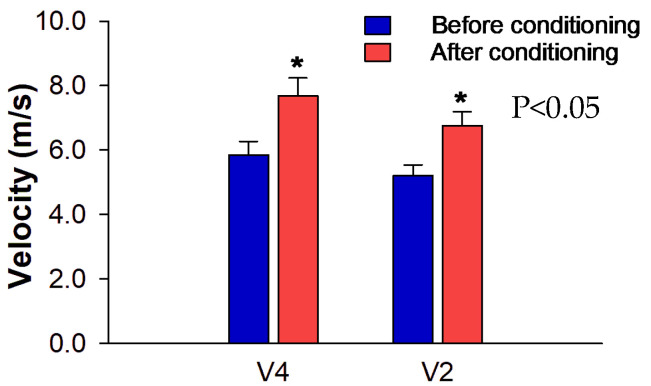
Mean ± standard deviation of V_4_ and V_2_ obtained in the incremental exercise test (IET). The graph represents the velocities reached in the test before and after the conditioning program. * Significant increase after conditioning compared to before conditioning (*p* < 0.05).

All 12 horses completed the six weeks of conditioning uneventfully. There were increases in both V_4_ (from 5.8 ± 0.4 to 7.6 ± 0.5 m/s) (*p* = 0.000) and V_2_ (from 5.2 ± 0.3 to 6.7 ± 0.4 m/s) (*p* < 0.0001) after the training program (Figure 4). The mean values of BW, age, thoracic circumference, and height are listed in Table 1. These data were measured in the first and second echocardiographic examinations, which had an interval of 5.5 months (during which the six-week conditioning was performed). After the conditioning, no increase in BW was observed (before: 309.67 ± 18.05 kg; after: 314.75 ± 25.15 kg) (*p* = 0.575). 

**Figure 5:**
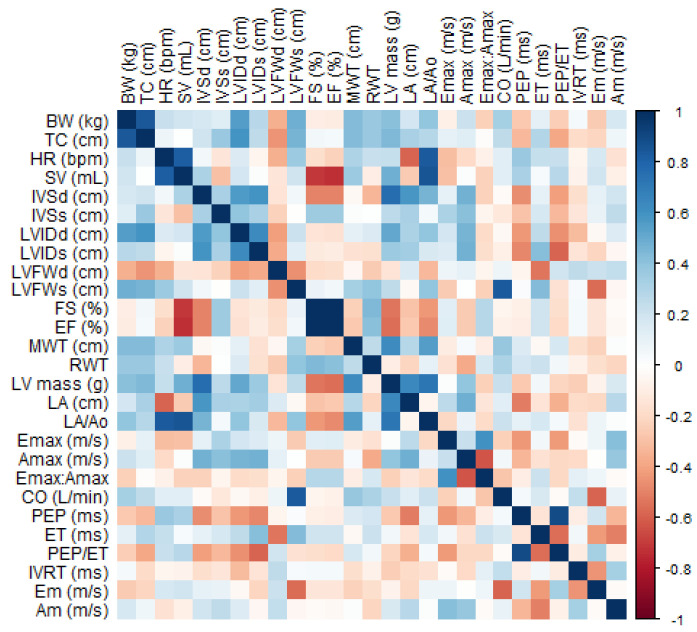
Heat map showing the correlation matrix between echocardiographic and body measurements. The color gradient indicates the correlation between BW, body weight; TC, thoracic circumference; and HR heart rate during echocardiography and other variables obtained and calculated from B-mode, M-mode, mitral flow Doppler (E_max_, A_max_, E_max_:A_max_), aortic flow Doppler (PEP, ET, PEP/ET) and tissue Doppler imaging of LVFW (IVRT, Em, Am). Refer to Table 2 and Table 3 for abbreviations.

In the qualitative assessment of the heart by echocardiography, no macroscopic differences were observed. As for the echocardiographic indices obtained in B- and M-modes, there were increases of 8.34% in LVIDd (*p* = 0.007) and 17% in LV mass (*p* = 0.011), whereas HR decreased (*p* = 0.003). No significant changes in LVFW, MWT, RWT, IVS, FS, EF, LA, Ao, and LA/Ao were verified (Table 2). The correlations between BW, thoracic circumference, and HR obtained by echocardiography and some selected cardiac variables are shown in Figure 5. In general, HR during the echocardiographic examination showed significant moderate negative correlations with thoracic circumference (r = −0.44), LA (r = −0.47), SV (r = −0.52), and aortic flow ET (r = −0.53). The other variables had no significant correlation with HR.

During the evaluation of the transmitral flow by pulsed-wave Doppler imaging, it was possible to observe a decrease in A_max_ (*p* = 0.047), while no changes in E_max_ and E_max_:A_max_ were noted (Table 3). The parameters of the aortic flow showed increases in ET (*p* = 0.029), VTI (*p* = 0.021), and SV (*p* = 0.042). No differences in PEP, ETI, PEP/ET, TTP, V_max_, CO, SI, and Vcf were found. In the second examination by tissue Doppler imaging, only ET increased (*p* = 0.011), with no significant changes being found in other periods and velocities (Table 3).

**Figure 6:**
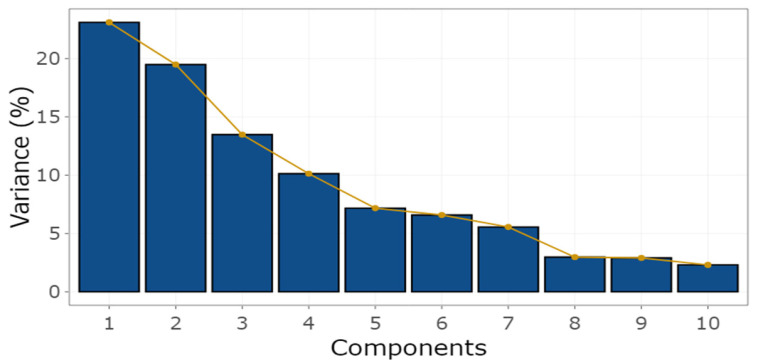
Percentage variation of principal components. The first seven components explained 84% of the variability of the echocardiographic variables. As can be observed, the degree of explainability was low from component eight onwards, justifying the presentation of only ten components.

**Figure 7:**
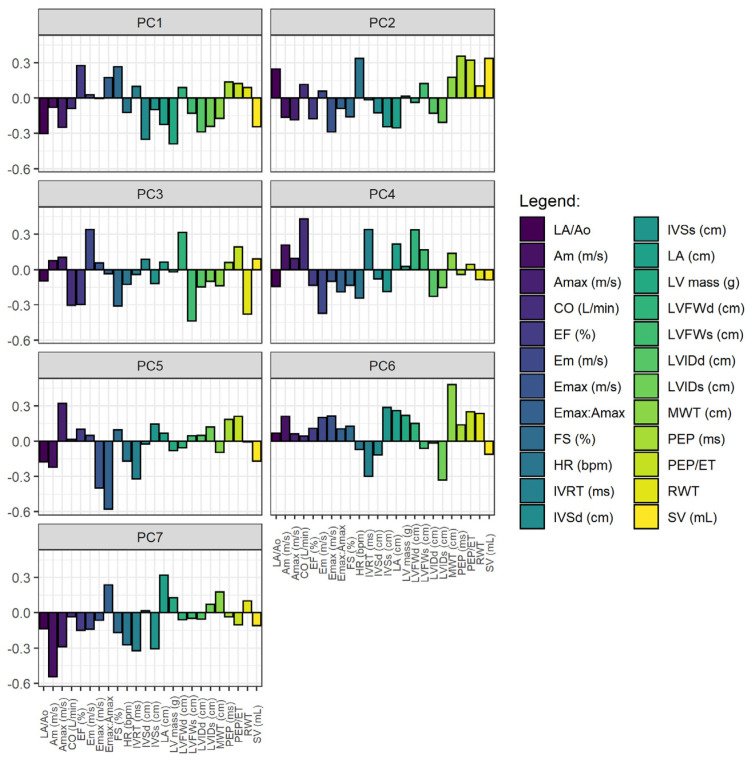
Principal components. In the two-dimensional image, it is possible to visualize the weight of each echocardiographic variable within each component. The variables selected for analysis were: HR, heart rate during echocardiography, and other variables obtained and determined in B- and M-modes with mitral flow Doppler (E_max_, A_max_, E_max_:A_max_), aortic flow Doppler (PEP, ET, PEP/ET), and tissue Doppler imaging of LVFW (IVRT, Em, Am). See legends in Table 2 and Table 3.

The principal component analysis reduced the dimensionality of 24 cardiac variables into seven components with eigenvalues > 1.0, explaining 84% of the total data variability (Figure 6). The contribution of each variable within its component can be seen in Figure 7.

## 4. Discussion

To the best of our knowledge, this is the first study to employ a lactate-guided physical conditioning program in naive young horses that did not perform any type of controlled exercise prior to the research in order to assess their cardiac changes by echocardiography. The animals were kept in a paddock; however, the voluntary activity had no relevant effect on fitness, given that it was not a regularly repeated exercise and the workload did not increase. The physical training employed was considered efficient since V_4_ increased by 31%, a value greater than that observed in other studies [18,21,31,32]. Furthermore, there was also an increase of 28.84% in V_2_. The conditioning program used in the present research proved to be suitable for young Arabian horses because of their enhanced aerobic capacity and absence of lameness, which represents a reduced risk of training-associated injury. It is also worth noting that the increases in both V_2_ and V_4_ were higher than those observed in a similar study [21], in which six-week training was applied to horses at 100% of V_2_ together with an IET every two weeks to adjust the training intensity to the new V_2_. In this study, V_4_ increased by 17% and the animals showed irritability and reluctance, which were associated with the onset of overtraining.

Given the difficulty of performing several IETs under practical conditions, the program tested in the present study can be successfully applied to young horses due to its safety and effectiveness in improving their endurance capacity. Nevertheless, the difference observed in the conditioning enhancement between the present work and that developed by Trilk et al. [21] may be related to the breed used. While the latter employed different breeds with a wide age range (4–20 years), the present study used a homogeneous group of young purebred Arabian horses, a breed that seems to have a greater oxidative capacity resulting from the high number of type I and IIa muscle fibers in relation to others [14]. On the other hand, when 90 day training was applied to purebred adult (8.6 ± 3.3 years old) Arabian horses at 80% of V_4_ with monthly velocity adjustment, no significant increase in V_4_ was observed in animals that did not receive supplementation, a parameter that was being evaluated in the referred study. However, the authors used horses that were kept inactive in a paddock for eight months [32]. Some degree of previous conditioning might have contributed to the insignificant increase in V_4_ since the intensity and period of conditioning were greater than those applied in the present study and the work conducted by Trilk et al. [21].

Even though the maximal lactate steady state (MLSS) was not determined in the current study, the increase in V_4_ was similar to that in V_2_–a parameter that approximates the MLSS velocity of horses [19]. V_4_ is not a reliable parameter to estimate the aerobic capacity of horses, as it overestimates the MLSS velocity [19,20,33]. The gold standard for estimating aerobic capacity is the MLSS test; however, it is not practical to perform this test with the number of animals used in this work. In addition, there is still no standardized alternative exercise test for appropriately assessing the anaerobic–aerobic threshold before and after a conditioning period [20].

In addition to improving the aerobic capacity, the conditioning program employed had a significant effect on the hearts of horses. Significant adaptations, such as increased LVIDd, LV mass, and SV, likely occurred due to the high preload caused by the expanded plasma volume resulting from physical training [34]. The increases in these variables corroborated the findings traditionally seen in humans [6,35] and dogs [36] after endurance training. Changes in LVID and LV mass are considered relevant, as they have strong positive correlations with aerobic capacity and are associated with better performance in thoroughbred horses [1]. The relationship between heart size and race performance was addressed in several studies [1,2,3,5]. Moreover, similar to what was found in racing breeds, higher LVIDd and SV in Arabian horses may lead to improved performance [4]. 

The fact that the classic “athlete’s heart”, characterized by increased thickness of the LVFW, did not develop may be attributed to the short duration of the conditioning program. Similarly, significant hypertrophy of the LVFW was also not observed in Arabian horses after training for four months [12] and in thoroughbred and standardbred horses after six months of training [10,11]. However, with the extension of the conditioning period, there could be an increase in the wall thickness of the left ventricle in order to reduce the tension in the myocardial wall caused by the enlargement of the ventricular chamber, according to Laplace’s law [35]. In addition to the duration, the intensity of the conditioning program also plays an important role in cardiac adaptation [11]. When comparing animals that performed training at different intensities, some authors observed higher LVID and LV mass in those trained at a higher intensity [2,4]. Furthermore, in contrast to the previously mentioned studies, wall hypertrophy was identified in thoroughbred horses after four and a half months of training [8]. 

It is known that LVIDd, LV mass, and MWT can increase with normal heart growth, as seen in standardbred horses [2,11]. Additionally, increases in both LA and LV were reported in purebred and crossbreed Arabians horses aged between four and six years [15]. Nonetheless, considering that the interval between echocardiograms was only 5.5 months, it is unlikely that the results of the present study can be solely attributed to normal growth. Another point to consider is that no expressive body development was verified during this period, according to the thoracic circumference and BW results. 

To differentiate conditioning effects from normal heart growth, a control group with animals of the same age and gender is required [4,7]. However, as it is difficult to find young untrained horses, only two studies used this resource [10,11]. Although differences in some echocardiographic measurements were found between trained and untrained groups before conditioning, cardiac adaptation was observed after six months of treadmill conditioning [10]. Nonetheless, no specific method was used to guide the training, and the changes were smaller than those observed in another study after 4.5 months of conditioning in a training center with horses of the same breed [8]. In another work, increases in BW, LVIDd, LV mass, and MWT were verified after six months of low-intensity conditioning in both trained and control groups due to normal growth [11]. Additionally, the percentage increase in ventricular diameter in young standardbred horses [2] after six months of conditioning was found to be greater than that reported by other researchers after ten months of training using animals of the same breed [7]. These divergences highlight the importance of carrying out a controlled study, as is the case in the present work, which despite not having included a control group, employed an individualized lactate-guided conditioning protocol according to the physical fitness of each animal. Therefore, this is the first study to evaluate cardiac adaptation in horses without any previous physical exercise after conditioning with standardized intensity.

Cardiac dimensions and function indices are influenced by HR, load, and contractility [23]. Despite the decreased HR in the second echocardiographic examination, it is unlikely that this parameter significantly influenced the results. In the first echocardiographic examination, the HR was slightly above the value commonly observed in horses at rest. This probably occurred because the horses were at the beginning of taming, which was carried out without the use of any physical exercise, and had not yet been fully adapted to the facilities and procedure. Thus, the HR obtained by echocardiography likely did not represent the true HR of the horses, and therefore could not be used to assess the effect of conditioning [2,8,10,11]. 

In humans, HR variations of 10% have minimal influence on cardiac dimensions and function indices [37]. In contrast, a study on the reliability and repeatability of echocardiographic measurements in horses indicated that an HR variation of 27% triggered the occurrence of significant changes in cardiac dimensions [38]. In addition, when comparing exercise stress echocardiograms with those obtained at rest, some researchers found differences in LVIDs and LVIDd only for HRs greater than 80 and 100 bpm, respectively [39]. Thus, the elevated LVIDd and SV observed in the present study may be explained by the volume overload caused by resistance training [40]. Moreover, among the cardiac variables that changed, only SV showed a moderate correlation with HR obtained by echocardiography.

With respect to SV, there are few studies in the literature on the effect of physical training in horses [41]. Additionally, the results reported and the forms of calculation are diverse, which can compromise a more accurate comparison [10,42]. The increase in SV at rest is a common finding in human athletes [13,40] and dogs with an absence of wall hypertrophy after endurance conditioning [37]. In the present work, although no wall hypertrophy was observed after conditioning, the SV increased by 26%, a value similar to that observed in young thoroughbreds after six months of track training [9]. When evaluating the physiological adaptations to conditioning after six and ten months, the researchers found a greater increase in aerobic capacity through maximum oxygen consumption ($\dot{V}$O_2max_) after six months of training. 

Likewise, a greater increase in SV was observed in treadmill-trained thoroughbred horses in the initial conditioning period, that is, after three months [10]. The main factor that led to an elevated $\dot{V}$O_2max_ was the SV, which was determined both by echocardiography and by the values obtained from the Fick principle (since other variables involved in oxygen transport did not change) [9]. This result reinforced the significant gain in conditioning obtained herein. 

Only a few studies in the literature evaluated the effect of physical training on both FS and EF at rest. Regarding FS, the results in human athletes and horses are conflicting. In horses, FS either increased [10], was reduced [2,8] or did not change [7], as occurred in the present study. It is known that these indices are influenced by loading, myocardial contractility, and HR [23]. In the present study, despite the increase in SV, HR during echocardiography decreased, which may have masked the change in FS, since this parameter showed a tendency to increase. On the other hand, in addition to the differences observed in HR at rest after training [8,10], the method used to determine FS and EF may also contribute to divergences in the results. In human athletes, there are also controversies about EF, given that this index has low sensitivity for the assessment of LV function in the presence of structural changes (as it does not consider geometric changes) [40]. After training, it is expected that systolic function would be preserved or increased [6]. However, as adaptation of LV contractility may not be detected due to the method used [40], further studies using more sensitive methods of assessing systolic function in horses are still needed to help differentiate pathological from physiological changes induced by exercise [43]. 

Even though the clinical application of tissue Doppler imaging for the assessment of LV systolic and diastolic functions in horses has been widely investigated [44], there is only one study in the literature evaluating its use in measuring cardiac adaptation to physical training. In the present study, no changes were detected in Em, Am, and Sm of the horses, corroborating the results reported in dogs after eight weeks of conditioning [36], despite the different type of measurement made, that is, short-axis versus apical plane images. In addition, while tissue Doppler imaging in horses assesses only the radial movement of the LV wall, it measures the longitudinal movement in dogs. The absence of changes in the present study and that by Restan et al. [36] may be related to the intensity and duration of the conditioning program. 

Unlike in the present study, when assessing the myocardial function of standardbred horses after ten months of conditioning using tissue Doppler imaging, some researchers observed a reduction in IVRT and an increase of 21.32% in the Sm wave of LVFW. The increased LVFW systolic velocity was attributed to the training effect since the animals developed an “athlete’s heart” [7]. As alterations in LV systolic function may occur after changes in the myocardial wall, this may justify why this function did not change in the present study, given the increased cardiac chamber and SV. Although tissue Doppler imaging is limited to the study of LVFW radial motion and, consequently, regional cardiac function, the proposed method is promising to understand cardiac adaptation in athletic horses [7].

In the “athlete’s heart” condition, LA remodeling tends to be common in humans [45] and dogs [36] after intense endurance training. In this sense, increases in both LV preload and volume are crucial for atrial remodeling since the volume of blood reaching the atrium rises along with atrial pressure in order to maintain the ventricular filling pressure [6,45]. Although in the present study there was an increase in the ventricular diameter, no significant change was found in LA. When comparing the endurance of Arabian horses classified as elite and non-elite, Sleeper et al. [4] observed no difference in atrial diameter despite an enlarged LV in elite animals. Furthermore, atrial enlargement seems to be dependent on the intensity and duration of conditioning in humans [6,45], which may explain the lack of alteration in horses at the beginning of their athletic career [15]. 

Even though there are numerous studies in medicine investigating the characterization and differentiation of physiological and pathological LA remodeling in humans, studies with horses are scarce. Only one study that evaluated the effect of conditioning in horses reported information on their LA, which increased after ten months [7]. Different from physiological remodeling, a pathological increase in LA elevates the risk of atrial fibrillation in elite athletes [45]. However, what is known about LA remodeling in horses is restricted to atrial fibrillation, which in horses may occur due to the chronic stretching of LA, triggered by valvular regurgitation [46]. The fact that horses may have regurgitation of multiple valves associated with adaptation to training [3] can make it difficult to clarify whether this species experiences physiological atrial remodeling induced by exercise, as is the case with humans.

Endurance sport athletes may have normal or supranormal diastolic function at rest with an increased contribution from early diastolic filling, which in turn elevates E_max_/A_max_ [6]. Likewise, after eight weeks of aerobic conditioning in dogs, increases in both LVIDd and E_max_/A_max_, were verified, along with a reduction in A_max_ of the transmitral flow [36]. Nevertheless, although A_max_ decreased in the current work, E_max_/A_max_ did not change. The lack of more consistent results on the improvement of diastolic function may be attributed to the short period of conditioning, the different HRs obtained between the tests due to the dependence of HR function indices, and the pre- and post-load and contractility conditions [23]. 

When a large number of variables is involved in a study, it is pertinent to select a smaller dataset [47]. In light of this, we opted to use principal component analysis, which proved capable of identifying and discriminating the echocardiographic variables that most accounted for the total variance. According to the contribution of each variable to the formation of the components, it was found that in component one, which explained 22% of the total variability, there was a greater contribution of LA, CO, LVIDd, LVIDs, MWT, LVFWd, LV mass, IVSd, SV, EF, FS, transmitral flow variables, E_max_:A_max_, and E_max_. This subset of variables expressed the maximum amount of information contained in the original variables and are therefore the most relevant to be explored in echocardiographic studies involving horse conditioning. Indeed, most of these variables changed after six and ten months of conditioning in horses [7,10] and eight weeks of conditioning in dogs [36].

In spite of the promising results, the present study had some limitations. The absence of a control group prevented the estimation of the total effect of conditioning on the cardiac adaptation of the horses. The fact that there may have been some influence of normal growth cannot be ruled out. Although both female and gelding horses were used, the number of geldings was small and the body development of the animals was homogeneous, pointing to an unlikely gender interference. However, further research should be developed to clarify whether the effect of training on the heart after conditioning at the same intensity differs between genders. Furthermore, considering that this study investigated the effect of a single conditioning program and only in purebred Arabian horses, different cardiac and conditioning adaptations may occur in other breeds submitted to different types of programs. Despite this, the aerobic feature of the conditioning protocol employed herein makes it a suitable foundation for any sport, since endurance improvement is a fundamental aspect regardless of the equestrian discipline. In addition, although cardiac assessment was performed at rest, the cardiac function can be better assessed after exercise by echocardiography due to the direct relationship between exercise intensity and oxygen demand [41]. Finally, an extension of the conditioning period is necessary since physiological changes can overlap pathological ones after intense and long-term training in human athletes.

## 5. Conclusions

Six weeks of aerobic training was considered efficient to improve the conditioning of horses and promote their cardiac adaptation, with an increase in ventricular diameter and stroke volume. Additionally, the conditioning program used proved to be safe for young horses, serving as a basis for future research.

## Figures and Tables

**Table 1:** Mean and standard deviation values of age, body weight, thoracic circumference, and height of purebred Arabian horses before and after a 5.5 month interval, during which six weeks of conditioning was performed.

Parameter	Before	After	*p*-Value
Age (months)	28.42 ± 3.75	34.42 ± 3.75	-
Body weight (kg)	312.17 ± 17.37	321.33 ± 19.13	0.232
Thoracic circumference (cm)	155.08 ± 3.78	158.25 ± 4.08	0.061
Height (m)	1.43 ± 0.02	1.44 ± 0.03	0.276

**Table 2:** Mean and standard deviation values of echocardiographic measurements of purebred Arabian horses obtained at rest, before and after the 5.5 month interval, during which six weeks of conditioning was performed.

B-Mode			
Parameter	Before	After	*p*-Value
HR (bpm)	42.24 ± 6.78	35.50 ± 2.73 *	0.003
LA (cm)	8.92 ± 0.68	9.16 ± 0.61	0.129
Ao (cm)	5.81 ± 0.44	6.09 ± 0.44	0.061
LA/Ao	1.56 ± 0.16	1.51 ± 0.17	0.275
**M-Mode**			
IVSd (cm)	2.15 ± 0.24	2.29 ± 0.20	0.089
IVSs (cm)	3.56 ± 0.25	3.75 ± 0,20	0.062
LVIDd (cm)	9.11 ± 0.72	9.87 ± 0.75 *	0.007
LVIDs (cm)	5.41 ± 0.71	5.59 ± 0.58	0.349
LVFWd (cm)	1.87 ± 0.27	1.84 ± 0.30	0.654
LVFWs (cm)	3.36 ± 0.22	3.40 ± 0.24	0.617
MWT (cm)	2.01 ± 0.19	2.07 ± 0.16	0.350
RWT	0.44 ± 0.04	0.42 ± 0.05	0.188
LV mass (g)	1610.86 ± 372.64	1886.90 ± 269.57 *	0.011
FS (%)	40.79 ± 4.04	43.40 ± 3.59	0.067
EF (%)	69.17 ± 4.79	72.01 ± 4.14	0.081

HR, heart rate; LA, left atrium; Ao, aortic diameter; LA/Ao, left atrium-to-aorta ratio; IVS, interventricular septum thickness; d, measurements at end-diastole; s, measurements at peak systole; LVID, left ventricular internal diameter; LVFW, left ventricular free wall thickness; MWT, mean wall thickness; RWT, relative wall thickness; LV mass, left ventricular mass; FS, fractional shortening; EF, ejection fraction. * Significant difference before and after conditioning (*p* < 0.05).

**Table 3:** Mean and standard deviation values obtained by pulsed-wave and tissue Doppler imaging of purebred Arabian horses at rest, before and after a 5.5 month interval, during which six weeks of conditioning was performed.

Transmitral Flow			
Parameter	Before	After	*p*-Value
E_max_ (m/s)	0.72 ± 0.08	0.68 ± 0.13	0.349
A_max_ (m/s)	0.41 ± 0.05	0.36 ± 0.05 *	0.047
E_max_:A_max_	1.78 ± 0.24	1.90 ± 0.44	0.433
**Aortic flow**			
PEP (ms)	96.39 ± 12.73	99.77 ± 23.34	0.437
ET (ms)	437.16 ± 48.32	472.89 ± 34.40 *	0.029
ETI (ms)	460.39 ± 46,72	492.41 ± 34.03	0.068
PEP/ET	0.22 ± 0.03	0.21 ± 0.06	0.604
TTP (ms)	130.97 ± 14.83	120.00 ± 17.62	0.113
V_max_ (m/s)	0.91 ± 0.09	0.96 ± 0.13	0.318
VTI (cm)	27.25 ± 4.42	31.58 ± 4.14 *	0.021
SV (mL)	444.60 ± 129.62	538.40 ± 100.43 *	0.042
CO (L/min)	18.47 ± 5.34	19.09 ± 3.65	0.682
SI (mL/kg)	1.42 ± 0.41	1.72 ± 0.26	0.050
Vcf (ms)	0.94 ± 0.15	0.92 ± 0.08	0.635
**Tissue Doppler imaging of LV free wall**			
PEP (ms)	117.11 ± 13.99	121.11 ± 16.18	0.383
ET (ms)	390.00 ± 58.47	434.22 ± 25.80 *	0.011
PEP/ET	0.31 ± 0.08	0.28 ± 0.04	0.220
IVRT (ms)	43.11 ± 21.96	41.33 ± 11.84	0.794
Em (m/s)	0.29 ± 0.05	0.28 ± 0.04	0.465
Am (m/s)	0.10 ± 0.01	0.09 ± 0.02	0.171
Sm (m/s)	0.11 ± 0.02	0.11 ± 0.01	0.627

E_max_, peak E wave velocity; A_max,_ peak A wave velocity; E_max_:A_max,_ E_max_-to-A_max_ ratio; PEP, pre-ejection period; ET, ejection time; ETI, ejection time index (corrected for HR); PEP/ET, PEP-to-ET ratio; TTP, time to peak; V_max,_ maximal velocity of the aortic flow; VTI, velocity time integral; SV, stroke volume; CO, cardiac output; SI, stroke index; Vcf, mean velocity of circumferential fiber shortening; IVRT, isovolumic relaxation time; Em, early-diastolic velocity; Am, late-diastolic velocity; Sm, ejection velocity. * Significant difference before and after conditioning (*p* < 0.05).

## Data Availability

The data that support the findings of this study are available upon request from the corresponding author.

## Supplementary Materials

The following supporting information can be downloaded at: https://www.mdpi.com/article/10.3390/ani13111800/s1, Table S1: Formulae used for the calculated parameters.
