# Supplementary material for: Cardiac Changes after Lactate-Guided Conditioning in Young Purebred Arabian Horses
Source: Animals (Basel). 2023 May 29;13(11):1800. doi: 10.3390/ani13111800 (PMC10252023; doi:10.3390/ani13111800)
Supplement: Supplementary file 1 [file animals-13-01800-s001.zip › animals-2339397-supplementary.pdf]

Table S1. Formulae used for calculated parameters.

Parameter	Equação
MWT (cm)	(LVFWd + IVSd)/2
RWT	(LVFWd + IVSd)/LVIDd
LV mass (g)	1,04 * ([LVIDd + LVFWd + IVSd]^3 – LVIDd^3) – 13,6
FS (%)	([LVIDd – LVIDs]/LVIDd) * 100
LVVd (cm^3)	(7 * [LVIDd^3]/2,4 + LVIDd)
LVVs (cm^3)	(7 * [LVIDs^3]/2,4 + LVIDs)
EF (%)	(LVVd –LVVs)/LVVd * 100
CSA	CSA = π * (Ao * 0.5)^2 (cm)
ETI (ms)	ET + (0,55 * HR)
SV (mL)	VTI * CSA
SI (mL)	SV/BW
CO (L/min)	HR * SV/1000
Vcf (mm/s)	(LVIDd-LVIDs)/(LVIDd * ET/1000)
